# Active Surveillance in *RET* Gene Carriers Belonging to Families with Multiple Endocrine Neoplasia

**DOI:** 10.3390/cancers13215554

**Published:** 2021-11-05

**Authors:** Alessandro Prete, Antonio Matrone, Carla Gambale, Valeria Bottici, Virginia Cappagli, Cristina Romei, Liborio Torregrossa, Laura Valerio, Elisa Minaldi, Maria Cristina Campopiano, Loredana Lorusso, Laura Agate, Eleonora Molinaro, David Viola, Teresa Ramone, Chiara Mulè, Raffaele Ciampi, Fulvio Basolo, Rossella Elisei

**Affiliations:** 1 Endocrine Unit, Department of Clinical and Experimental Medicine, University Hospital of Pisa, Via Paradisa 2, 56124 Pisa, Italy; alessandro.prete22@gmail.com (A.P.); antonio.matrone@med.unipi.it (A.M.); gambalecarla@libero.it (C.G.); v.bottici@ao-ao-pisa.toscana.it (V.B.); virginia.cappagli@med.unipi.it (V.C.); cristina.romei@unipi.it (C.R.); lau.val@hotmail.it (L.V.); elisa.minaldi@med.unipi.it (E.M.); cristina.campopiano@med.unipi.it (M.C.C.); lorussoloredana@hotmail.it (L.L.); laura.agate@virgilio.it (L.A.); e.molinaro@ao-pisa.toscana.it (E.M.); violadavid@hotmail.it (D.V.); teresa.ramone@hotmail.it (T.R.); c.mule@studenti.unipi.it (C.M.); raffaele.ciampi@unipi.it (R.C.); 2 Pathology Unit, Department of Surgical, Medical, Molecular Pathology and Critical Area, University of Pisa, 56124 Pisa, Italy; l.torregrossa@ao-pisa.toscana.it (L.T.); fulvio.basolo@med.unipi.it (F.B.)

**Keywords:** medullary thyroid cancer, calcitonin, MEN2, gene carriers

## Abstract

**Simple Summary:**

MEN2 has a very high penetrance for the development of medullary thyroid cancer. However, intra- and inter-familial variabilities have been described. Accordingly, in this precision medicine era, a personalized approach should be adopted in subjects harboring *RET* mutations. In these subjects, we showed that thyroid surgery could be safely timed according to basal and stimulated calcitonin, especially in children who can reach adulthood, avoiding the risks of thyroid surgery and decreasing the period of a long-life hypothyroidism treatment.

**Abstract:**

Multiple Endocrine Neoplasia 2 (MEN2) is a hereditary cancer syndrome for developing medullary thyroid cancer (MTC) due to germline mutations of *RET* gene. Subjects harboring a germline *RET* mutation without any clinical signs of MTC are defined as gene carriers (GCs), for whom guidelines propose a prophylactic thyroid surgery. We evaluate if active surveillance of GCs, pursuing early thyroid surgery, can be safely proposed and if it allows safely delaying thyroid surgery in children until adolescence/adulthood. We prospectively followed 189 GCs with moderate or high risk germline *RET* mutation. Surgery was planned in case of: elevated basal calcitonin (bCT) and/or stimulated CT (sCT); surgery preference of subjects (or parents, if subject less than 18 years old); other reasons for thyroid surgery. Accordingly, at *RET* screening, we sub-grouped GCs in subjects who promptly were submitted to thyroid surgery (Group A, *n* = 67) and who were not (Group B, *n* = 122). Group B was further sub-grouped in subjects who were submitted to surgery during their active surveillance (Group B1, *n* = 22) and who are still in follow-up (Group B2, *n* = 100). Group A subjects presented significantly more advanced age, bCT and sCT compared to Group B. Mutation *RET*^V804M^ was the most common variant in both groups but it was significantly less frequent in Group A than B. Analyzing age, bCT, sCT and genetic landscape, Group B1 subjects differed from Group B2 only for sCT at last evaluation. Group A subjects presented more frequently MTC foci than Group B1. Moreover, Group A MTCs presented more aggressive features (size, T and N) than Group B1. Accordingly, at the end of follow-up, all Group B1 subjects presented clinical remission, while 6 and 12 Group A MTC patients had structural and biochemical persistent disease, respectively. Thank to active surveillance, only 13/63 subjects younger than 18 years at *RET* screening have been operated on during childhood and/or adolescence. In Group B1, three patients, while actively surveilled, had the possibility to reach the age of 18 (or older) and two patients the age of 15, before being submitted to thyroid surgery. In Group B2, 12 patients become older than 18 years and 17 older than 15 years. In conclusion, we demonstrated that an active surveillance pursuing an early thyroid surgery could be safely recommended in GCs. This patient-centered approach permits postponing thyroid surgery in children until their adolescence/adulthood. At the same time, we confirmed that genetic screening allows finding hidden MTC cases that otherwise would be diagnosed much later.

## 1. Introduction

Multiple Endocrine Neoplasia 2 (MEN2) is an hereditary cancer syndrome characterized by the development of medullary thyroid cancer (MTC), variably associated with other endocrine neoplasia, such as pheochromocytoma and primary hyperparathyroidism [1,2,3]. MEN2 is an autosomal dominant disease with a very high penetrance due to missense gain-of-function mutation of the *RET* gene (Rearranged during Transfection) [4,5]. Germline *RET* mutation is present in about 99% of familial and about 6.0% of apparently sporadic cases of MTC [6]. Accordingly, germline *RET* screening must be offered to all patients with MTC and, if positive, all first-degree relatives should be screened [7,8]. Subjects harboring a germline *RET* mutation without any clinical signs of MTC are defined as Gene Carriers (GCs) [8].

In the case of a GC, guidelines propose a prophylactic thyroid surgery as “the removal of the thyroid before MTC develops or while it is clinically unapparent and confined to the gland” [8]. Its timing is essentially based on subject *RET* mutation and age; in cases of *RET* mutation at highest risk (*M918T*) surgical therapy must be performed within the first year, in cases at high risk (*C634F/G/R/S/W/Y* and *A883F*) the timing of thyroidectomy can be based on serum calcitonin (CT). However, in any case before 5 years and in cases at moderate risk (other mutations), basal and stimulated CT (bCT and sCT) should guide thyroid surgery timing [8]. This latter suggestion is not always followed in the real clinical world and several centers still follow the indication to operate immediately after the *RET* screening, warning against the use of serum CT in this clinical scenario [9]. 

By many years, in the case of GCs harboring high and moderate risk mutations, in our center we are performing an active surveillance by timing the thyroid surgery on bCT and sCT levels, regardless of *RET* mutation and age, pursuing an early, instead of a prophylactic, thyroid surgery [10]. The main reasons are related to both the higher risk of surgical complications in children, particularly permanent hypoparathyroidism that implies long-life therapy [11], and to the need of early medication with levothyroxine during childhood and adolescence in subjects who actually have normal thyroid function. 

In this study, we evaluated if an active surveillance with an early thyroid surgery can be safely proposed in *RET* GCs and for how many years the surgery could be safely delayed in children. Moreover, we looked also at the relevance of genetic screening in finding hidden MTC cases that, otherwise, would be diagnosed much later. 

## 2. Materials and Methods

### 2.1. Subjects

After 1993, we performed *RET* genetic screening in all patients with diagnosis of MTC, either familial or apparently sporadic and, if positive, to all their first-grade relatives [6]. 

All adult patients signed informed consent to perform *RET* genetic screening. Parents or guardians signed the informed consent in the case of subjects less than 18 years of age. As per the policy of the University Hospital, all patients provided written informed consent to both the genetic screening and the use of their clinical and biochemical data for scientific purposes.

### 2.2. Clinical Evaluation

We evaluated GCs by using clinical, biochemical (i.e., bCT and sCT (pentagastrin (Pg) stimulation test up to 2013, and then calcium (Ca) stimulation test, as elsewhere described [12]), urinary metanephrine and normetanephrine, serum PTH, calcium and 25-hydroxyvitamin D (25[OH]D) and imaging examinations (i.e., neck and abdominal ultrasound and whenever necessary abdominal MRI).

### 2.3. Surgery Criteria

According to the most recent advances carried out by Elisei et al. [10], in our center the surgical treatment for GCs, independently from the type of germline mutation, is planned according to the following criteria:

(1) elevated bCT (i.e., higher than upper limit of normal range) and/or positive stimulation test;
(2) subjects (or parents when subjects were under the age of 18) who specifically asked for immediate surgery;
(3) other reasons for thyroidal surgery (e.g., Graves disease or symptomatic goiter).

Otherwise, patients without any of the above-mentioned criteria were followed every 6–12 months with clinical, biochemical, and morphological assays (namely neck ultrasound) as previously described.

### 2.4. Post-Surgery Follow-Up

Four/six months after surgery, all patients were submitted to biochemical analysis (bCT and, if necessary, Pg or Ca stimulation test for CT) and neck ultrasound. Whenever indicated, other imaging (e.g., CT scan, MRI etc.) were performed.

### 2.5. RET Genetic Analysis

*RET* genetic screening has been performed on DNA extracted from the blood of MTC patients and of their relatives according to a procedure previously reported [6]. MTC patients have been screened for the presence of *RET* mutations in exons 5, 8, 10, 11, 13, 14, 15, and 16 while relatives of *RET* positive index cases have been analyzed only for the presence of the mutation identified in their family. Actually, genomic DNA is amplified using KAPA2G Fast HotStart PCR Kit (Sigma-Aldrich, Saint Louis, MI, USA) in a final volume of 20 μL with 0.5 pmoli/μL of each primer and using a SimplyAmp thermal cycler (Thermofisher, Waltham, MA, USA). Amplification cycle is performed with an initial step of 95 °C for 2 min, followed by 35 cycles of 95 °C for 15 s, 60 °C for 15 s and 72 °C for 15 s. A final extension at 72 °C for 7 min was performed at the end of the amplification protocol. Sequence analysis was performed, and has been reported on previously. Primers’sequence can be provided upon request. Sequence reactions are performed according to the Sanger method using an ABI Prism 3130XL genetic analyzer (Thermofisher, Waltham, MA, USA).

### 2.6. Laboratory Evaluation

In the last 25 years CT measurement has been performed using two immunometric assays (ELSA-hCT, Cis-BioInternational, Gif sur Yvette, France, functional sensitivity 10.0 pg/mL, from 1993 to 2013 and chemoluminescent immunometric Immulite, Siemens Healthcare Diagnostic Products Ltd., Lianberis, Gwynedd LL55 4EL, UK, with analytic sensitivity 2.0 pg/mL reference values of up to 18.2 pg/mL for women and 11.5 pg/mL for men, from 2014 to the present).

### 2.7. Histopathology

All the specimens were submitted to routine pathological procedure and were reviewed by two pathologists (LT, FB). Briefly, the surgical specimens were fixed in 10% buffered formaldehyde and embedded in paraffin, and then 4-mm-thick sections were cut and stained with hematoxylin & eosin (H&E). For immunohistochemistry, paraffin sections (3–5 mm) were dewaxed in xylene, dehydrated through graded alcohols, and processed using the diaminobenzidine detection system. All of the immunohistochemical analyses for calcitonin were performed automatically using the Ventana Benchmark^®^ immunostaining system (Ventana Medical Systems, Tucson, AZ, USA) and a rabbit monoclonal primary antibody direct against calcitonin polypeptide (Ventana Medical Systems, clone SP17; dilution 0.56 µg/mL). 

Usually, on routine H&E stained-slides the “neoplastic” or “primary” CCH is easily identified by the presence of clusters of intrafollicular C-cells, composed of cells with mild or moderate cellular atypia, resembling those identified in an MTC [13]. According to the last edition of WHO Classification of Tumours of Endocrine Organs [14] the diagnosis of “primary” CCH is encountered when >6–8 C cells per cluster in several foci with >50 C cells per low power field are identified. Immunostaining for CT was performed in all cases to confirm the recognition of C-cells. Histologically, the main difference between the “primary” CCH and the microfocus of MTC is represented by extension of C cells through the basement membrane into the surrounding thyroid interstitium or when a desmoplastic stromal reaction surrounding the infiltrating neoplastic cells is evident [15].

### 2.8. Statistical Analysis

Statistical analysis was performed using Kruskal–Wallis, Mann–Whitney, *t* tests, ROC curves, univariate and multivariate regression analysis, according to the variables to be analyzed, using IBM SPSS Statistics (Armonk, NY, USA) for Macintosh, Version 25.0. A *p* value less than 0.05 was considered statistically significant.

## 3. Results

### 3.1. Study Groups: Epidemiological, Biochemical and Genetics Data

*RET* genetic screening allowed us to discover 189 GCs in 84 families. At first clinical evaluation, after the screening, we distinguished two groups of GCs: those who already met surgery criteria (*n* = 67, Group A) and those who did not (*n* = 122, Group B). Epidemiological, biochemical and US data of Group A and B, are reported in Table 1. Group A subjects were significantly older than Group B (median 44 vs. 18 years) (*p* < 0.0001). As expected, at *RET* genetics screening, Group A subjects presented significantly higher bCT (median 24 ng/L vs. below functional sensitivity) as well as sCT (median 276.5 vs. 10.6 ng/L) compared to Group B (*p* < 0.0001). US scan identified thyroid nodule in 71.2% (37/52) of Group A subjects and in 22.1% (23/104) of Group B (*p* < 0.0001) (Table 1).

**Figure 1:**
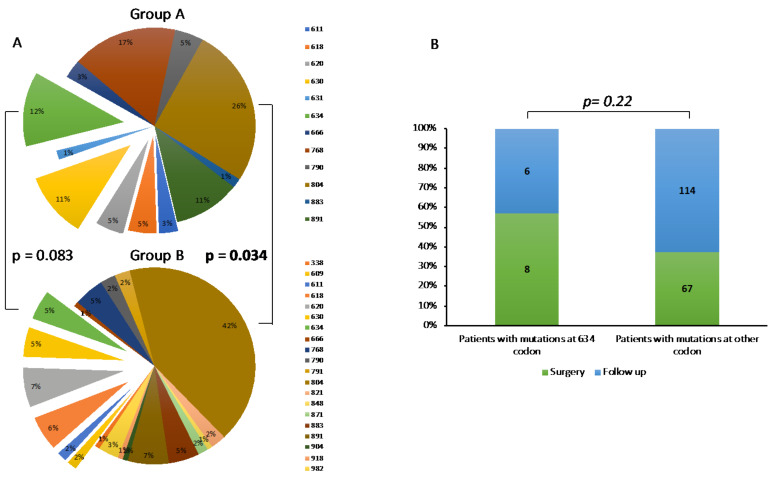
(**A**) Genetic landscape of Group A (upper graph) and B (lower graph). (**B**) Rate of patients submitted to surgery or follow-up, according to RET mutations (634 codon vs. other codons).

We analyzed the genetic landscape of Group A and B. In agreement with our previous report [6], we confirmed that mutations occurring at 804 codon were the most common mutations in both groups, although they were significantly less frequent in Group A than B (26% vs. 42%, *p* = 0.034) (Figure 1A). Otherwise, we observed that mutations occurring at 634 codon were substantially, although not significantly, more frequent in Group A than B (12% vs. 5%, *p* = 0.083) (Figure 1A). Accordingly, at first evaluation, 57% of patients with *RET^C634X^* mutation presented the criteria for surgery while only 37% with other mutations presented these criteria, although this difference was not statistically significant, probably due to relatively low number of subjects with *RET^C634X^* mutation (*n* = 14) (*p* = 0.22) (Figure 1B).

### 3.2. Follow-Up in Group B

**Figure 2:**
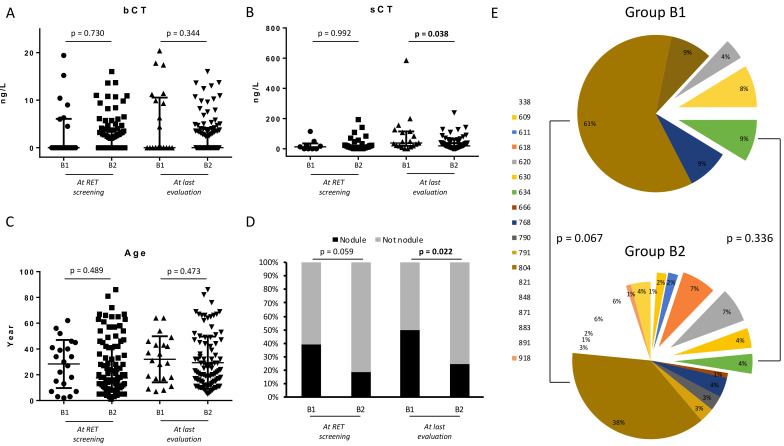
(**A**,**B**) basal CT (bCT) and stimulated CT (sCT) in Group B1 and B2 at *RET* screening and at last evaluation. (**C**) Age of subjects of Group B1 and Group B2 at *RET* screening and at last evaluation. (**D**) Rate of nodule at neck ultrasound in subjects of Group B1 and Group B2 at RET screening and at last evaluation. (**E**) Genetic landscape of subjects of Group B1 and Group B2.

After the *RET* genetics screening assessment, Group B subjects were followed every 6–12 months. During their follow-up, 22/122 (19%) subjects were submitted to surgery (Group B1) after a median time of 1.6 years (IQR 1.1–3.6, range 1.1–10.3 years) and 100/122 (81%) patients are still in follow-up (Group B2) after a median time of 2.9 years (IQR 0.9–6.3, range 0.1–21.8 years). We analyzed epidemiological, biochemical, and US-features of GCs of Groups B1 and B2 both at *RET* screening and last evaluation (either before surgery in Group B1 or at the end of follow-up in Group B2). At *RET* screening evaluation, Groups B1 and B2 subjects did not differ for age, bCT, and sCT (Figure 2). Otherwise, at the last evaluation, Group B1 subjects presented significantly higher levels of sCT compared to Group B2 (median 38 vs. 20 ng/L, respectively, *p* = 0.035), whereas bCT and age were not different (Figure 2). At US scan, thyroid nodules were substantially more frequent in Group B1 than Group B2 at *RET* screening evaluation (42%, 8/19 vs. 17.6%, 15/85; *p* = 0.059) and significantly at last evaluation (50%, 10/20 vs. 25%, 23/92; *p* = 0.022) (Figure 2D). Figure 2E summarized genetics landscapes of both groups. Mutations occurring at 804 codon were substantially, although not significantly, more frequent in Group B1 (61%, 14/23) than B2 (38%, 37/98) (*p* = 0.067). Mutations at 634 codon did not differ between the two groups. 

### 3.3. MTC in Group A and B1: Anatomopathological Features, Prognosis and Surgical Complications

**Figure 3:**
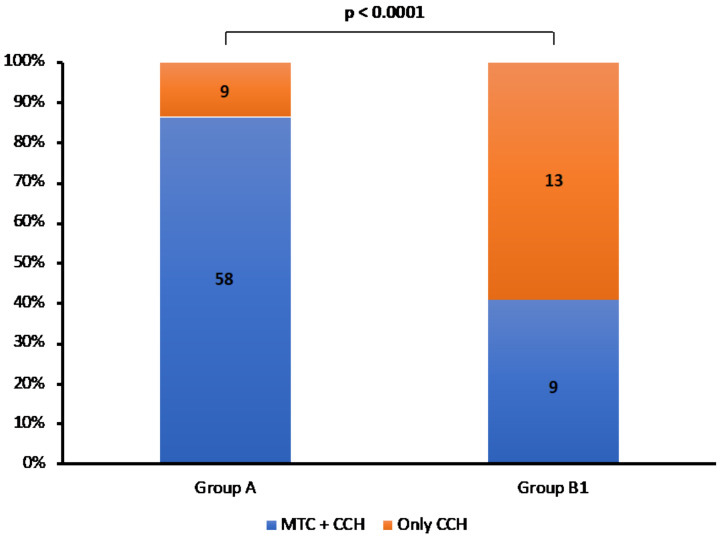
Rate of MTC + CCH and only CCH in Group A and B1 patients.

At histology, all cases showed MTC foci and/or CCH. We compared anatomopathological features between Groups A and B1 and we found that MTC foci ± CCH was significantly more present in Group A (58/67, 86.7%) than B1 (9/22, 40.9%), in which the CCH alone was prevalent (Figure 3) (*p* < 0.0001).

Among those patients who had MTC foci, Group A patients had MTC foci significantly larger than Group B1 (median 0.65 vs. 0.40 cm, *p* value = 0.036). At variance, MTC multifocality and bilaterality were not different in Groups A and B1 (Table 2). We, therefore, analyzed TNM classification system in patients of Group A and B1 with MTC (67 patients). Although most of the MTC patients belonging to Group A had T score of 1 (51/58, 88%), a significant portion (7/58, 12%) had T score > 1, whereas all MTC patients of Group B1 had a T score of 1. Lymph node metastasis occurred in 21 patients of Group A, while they did not occur in Group B1 patients (*p* = 0.045). In the case of lymph-node metastasis, they occurred in 15/21 patients (71.4%) in central and in 6/21 in latero-cervical (28.6%) compartments (Table 2). At the time of surgery, only one case of Group A presented metastasis spread to lungs, liver, and bones. All MTC patients of Group B1 experienced clinical remission during the follow-up after surgery (median 4 years, IQR 2–7 years, intervals 3–153 months), while 6/67 (9%) and 12/67 (18%) MTC patients of Group A had structural and biochemical persistent disease, respectively, during their follow-up (median 6.5 years, IQR 2.25–13 years, 3–311 months) (Table 2). All patients of both groups with CCH were cured at the data lock of this study (median follow up 4.6 years, IQR 2.5–11, 1–178) as assessed by undetectable levels of both bCT and sCT.

About surgical complications, they were observed in 15 (22.4%) patients of group A and in only one (6.3%) of group B1 (*p* = 0.059). Among group A patients, 14 of them presented only hypoparathyroidism and one patient both recurrent laryngeal nerve injury and hypoparathyroidism. Patient of group B1 developed only hypoparathyroidism.

### 3.4. Follow-Up of GCs under the Age of 18

**Figure 4:**
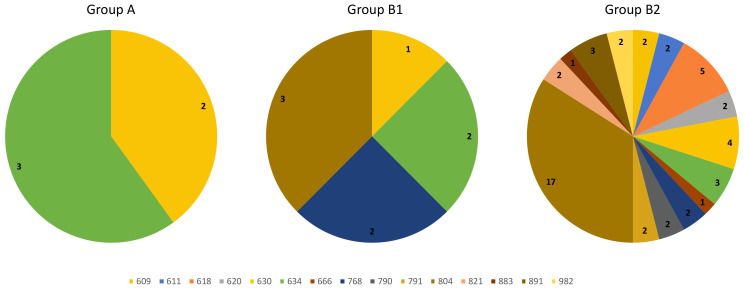
Genetic landscape of subjects younger than 18 years belonging to Group A, B1 or B2.

Looking at GCs younger than 18 years at the time of *RET* genetic screening, we had a total of 63 subjects. Applying the aforementioned surgery criteria, 5/63 patients were submitted to surgery after first evaluation (belonging to Group A), 8/63 during their follow-up (belonging to Group B1), while 50/63 individuals are still on follow-up (belonging to Group B2). At *RET* genetics screening, there was not any difference between age of subjects belonging to Group A (median age 10 years old, IQR 6–14, intervals 5–15 years), to Group B1 (median age 7 years old, IQR 3–12.5, intervals 2–15 years) or to Group B2 (median age 8 years old, IQR 5–13, intervals 1–17 years). Otherwise, as expected, mutations occurring at 634 or cysteine codon were significantly more common in group A, although present also in Group B1 and B2 (*p* = 0.001 for 634 codon and *p* = 0.021 for cysteines); likewise, *RET^C634X^* mutations were substantially more common in Group B1 than Group B2 (*p*= 0.075), whereas mutations occurring at cysteine or 804 codons did not differ in Group B1 and Group B2 (*p* = 0.935 and *p* = 0.847, respectively) (Figure 4).

**Figure 5:**
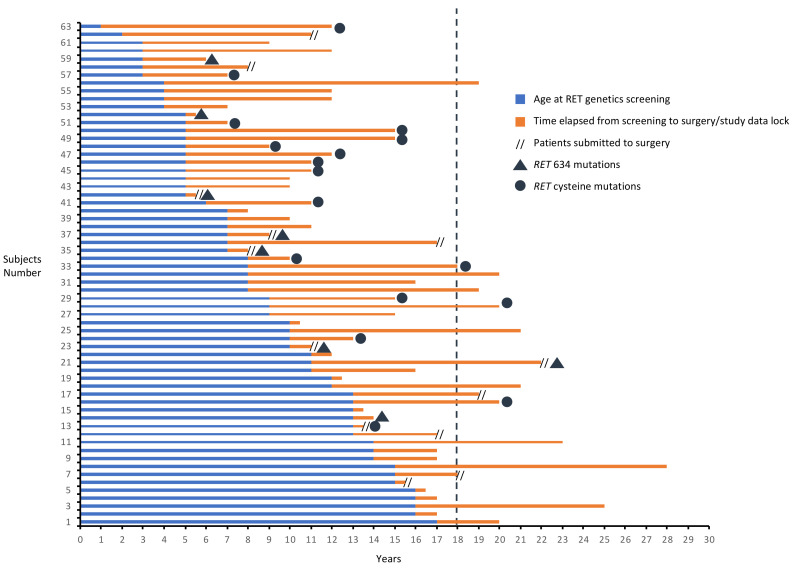
Genetic landscape, and duration of follow-up, of GCs younger than 18 at the time of *RET* genetic screening of Group A, B1 and B2.

Surgery was performed after a median time of 5 months (IQR 4–7, intervals 4–7 months) in subjects of Group A and of about 3 years (IQR 1.6–9.3, intervals 1.6–10.3 years) in subjects of Group B1. So far, only 11/63 (17.5%) patients have been operated during childhood and/or adolescence. At the study data lock, a total of 15/58 (25.9%) GCs who did not immediately meet the criteria for surgery reached the age of 18 and two of them have been operated at 18 and 22 versus 15 and 11 years at screening. Among Group B1 patients, at time of surgery, two of them (one patient with *RET^C634Y^* and one with *RET^V804M^*) became older than 18 years, one reached 18 years (with *RET^V804M^*) and two older than 15 (two patients with *RET^E768D^*) (Figure 5). Only one patient (age at surgery of 17 years) developed a surgical complication (hypoparathyroidism). Among patients who are still in follow-up (*n* = 50), (median time of 5 years, IQR 3–9, intervals 1–15 years) at study data lock, 12 patients became older than 18 years and 17 older than 15 years (Figure 5).

## 4. Discussion

Oncology was radically revolutionized by screening of hereditary cancer diseases, diminishing the rate of patients with advanced disease at diagnosis and their mortality [16]. According to genetic and clinical characteristics of each hereditary disease, several approaches may be proposed: prophylactic surgery of involved organ, regular biochemical and/or morphological screening to promptly identify an arising neoplasia, and chemoprevention to hinder cancer development [16]. In MEN2, the suggested approach swings between the prophylactic surgery (in case of highest and very high risk *RET* mutations) and the regular follow-up (in case of moderate risk *RET* mutations), while chemoprevention has not gained space so far [8,17]. If the surgical approach must be proposed before 1 year of age in patients harboring *RET^M918T^*, in case of other *RET* mutations a personalized approach should be persuaded [8,18].

In this prospective study looking at 189 GCs with high and moderate risk *RET* mutations, we showed that thyroid surgery might be safely planned following bCT and sCT. In particular, GCs who were submitted to surgery after a regular follow-up (Group B1) did not experience neither lymph-node nor distant metastasis, and neither biochemical nor structural persistence was observed, at least at study data lock (median follow-up 4 years). Although the median follow-up is rather short, we should consider that all these patients showed a negative CT stimulation test at 3–6 months after surgery, which implies a negligible risk of possible recurrence [19].

The disease status of GCs who already had the criteria for surgery at the time of *RET* genetic screening (Group A) was indeed more advanced with 21/67 (31.3%) patients having lymph-node and 1/67 (1.5%) distant metastasis. Despite the prompt thyroidectomy and lymphadenectomy, 9.0% and 18% of them had structural and biochemical persistent disease, respectively, after a median follow-up of 6.5 years. However, if we consider that the percentage of MTC patients with lymph-node metastasis and/or distant metastasis in big series of MTC is around 45.1–53% and 10–11.4%, respectively [19,20,21], our findings demonstrate that, even in already affected GCs, the *RET* genetic screening can anticipate the diagnosis when the MTC is still clinically silent. This evidence confirmed that *RET* genetic screening should be offered and solicited to all first-degree relatives of patients with MEN2, as recommended by MTC guidelines [7,8].

We also found a relevant difference of both disease stage and outcome between group A and B1, demonstrating that timing surgery according to the increase of bCT and sCT allows performing an early, but not prophylactic, thyroid surgery that is still safe, since all patients in group B1 were cured at the time of data lock but also justified since microfoci of MTC were already present in more than 40% of cases [10].

Recently, Machens et al. showed that the risk of lymph-nodes metastasis in patients harboring *RET* germline mutations increased by age and by *RET* risk category (e.g., low-moderate vs. moderate-high and high risk) [22]. In our series, patients of Group A, were effectively older than those of Group B while no significant differences were found in the type of RET mutations except for the fact that V804M was more frequent in Group B. This finding confirms the role of the advanced age in the development of the disease but reduces the impact of the type of *RET* mutation. New evidence showed that RETV804M mutation harbors a moderate risk of MTC development [23], although in our cohort this risk seems to not be negligible. Although age and *RET* mutation seem to be two milestones of MEN2 phenotypic variability, it is far long to be completely enlightened and inter- and intra-familial variability has been shown by many authors [24,25,26,27,28]. Accordingly, patients in Groups B1 and B2 did differ neither in age nor in *RET* genetics while they differ in the biological behavior of the tumor whose growth was faster in Group B1. These data argued that MEN2 genotypic-phenotypic relation is less stiff than imagined in the past and might be influenced by other factors: genetics (e.g., unbalanced expression of mutant and wild *RET* gene), epigenetics (e.g., DNA methylation, histone modification, or chromatin remodeling) and non-genetics (e.g., environmental factor) [29]. In this nebulous scenario, *RET* mutation and age should certainly guide clinical decisions, but these data argued that each clinical management must be individualized, and thyroid surgery should be timed according to bCT and sCT, avoiding prophylactic surgery that is necessarily followed by the medicalization of the patients and, sometimes, by surgical complications, especially in children.

Using this approach, 15 out 58 patients, who were younger than 18 years of age at the time of screening, reached adulthood without thyroid surgery, postponing the beginning of a long-life therapy with levothyroxine (LT4). LT4 is the only current recommended therapy for patients undergone to total thyroidectomy, both in adults and children [7,30]. However, although a biochemical euthyroidism is generally reached, LT4 seems to do not guarantee an euthyroidism state in all tissues [31,32]. In addition, biochemical features in athyreotic patients seem to be different from those in euthyroid ones, as demonstrated by Gullo and colleagues, who showed that athyreotic patients treated with LT4 had higher fT4 and lower fT3 levels than euthyroid control and about one third of them had lower than reference fT4/fT3 ratio [33]. It is unknown if this not physiological thyroid state might play a role in children growth.

Transient or permanent disruption of calcium metabolism may occur after thyroid surgery in more than 25% and 5% of patients, respectively [34]. De Jong and colleagues collected clinical and biochemical data of 106 children (younger than 18 years) submitted to thyroid surgery and described a hypocalcemia at discharge in 49.3% and at 6 months 21.7% of them [35]. The higher risk of hypoparathyroidism in children compared to adult was confirmed by other authors [36,37], in particular in those younger than 3 years old [37]. In our cohort of GCs younger than 18 years who were submitted to surgery (13), only one patient (1/13, 7.7%) is experiencing a permanent hypoparathyroidism. Otherwise, this risk seems to be minimized in high-volume facilities [38,39], especially in patients who do not need central neck dissection [40]. Accordingly, in order to minimize this risk, GCs should be referred to surgical centers experienced in pediatric surgery for thyroid cancer. In this scenario, safely postponing thyroid surgery across the childhood could be a winning choice.

A recent review observed that subjects who experienced cancer diagnosis during their childhood seemed to be at higher risk of impaired psychological development [41], as well as manifestations of anxiety, depression, inattention, and antisocial behavior [42]. Adult survivors of childhood cancer were described to be at higher risk of depression and anxiety symptoms, even many years after the end of therapies [43]. At the same time, adults with a history of cancer during childhood presented poorer social outcomes, such as the capacity of living independently or psychosexual milestones in both females and males [43,44,45]. According to this evidence, the psychological impact of thyroid surgery should be carefully evaluated in children, especially after this demonstration that by taking children in active surveillance once a year and postponing the thyroidectomy to an early phase of the disease development their outcome is still favorable like that obtained with prophylactic thyroidectomy.

Finally, this approach requires an adherence of GCs (and their parents, in case of children) to regular assessments, which could represent a limitation of this active surveillance approach in our mobile society. However, like in other chronic conditions, patient education and participation are vitally important [46,47]. Subjects with RET mutations (and their parents) must be highly informed about the advantages and the disadvantages of this approach. In this highly personalized approach, each GC must not be a passive character but an active and collaborative player and social and psychological needs of each subject should be considered.

## 5. Conclusions

Our data showed that an active surveillance pursuing an early thyroid surgery, based upon bCT and sCT, could be safely recommended in high and moderate risk *RET* GCs, both adults and children, thus reducing the lifespan of medicalization and the risk of surgical complications. This is particularly desirable in children and is independent from the type of *RET* mutation even if those with a high risk mutation likely will reach the need to be operated earlier than those with moderate risk mutations. Moreover, we confirmed that genetic screening allows finding hidden MTC cases that otherwise would be diagnosed much later.

## Figures and Tables

**Table 1:** Epidemiological, biochemical and US data of Group A and B. bCT: basal calcitonin, sCT: calcitonin upon pentagastrin or calcium stimulation test, US: ultrasound, BFS: below functional sensitivity.

Determinants		Group A (*n* = 67)	Group B (*n* = 122)	*p* Value
Follow-up (years) median (IQR, intervals)		7 (1.5–12.5, 0.3–26)	3.6 (0.8–6.5, 0.08–21.8)	0.0001
Male: Female (number of patients)		31:36	57:65	0.952
Age at *RET* screening (years) median (IQR, intervals)		44.0 (30–56, 5–80)	18.0 (8–41.3, 1–86)	<0.0001
bCT at *RET* screening (ng/L) median (IQR, intervals)		24.0 (0–245, 0–33571)	BFS (BFS-3.9, BFS-19.4)	<0.0001
sCT at *RET* screening (ng/L) median (IQR, intervals)		276.5 (38–1175, 0–17810)	10.6 (BFS-21.4, BFS-193)	<0.0001
US assessment at *RET* screening	Presence of at least one nodule	71.2%	22.1%	<0.0001
	Negative	28.8%	77.9%	

**Table 2:** Histopathological features of MTCs of Group A and B1 patients.

Histopathological Features	Group A *n* = 58/67 (86.7%)	Group B1 *n* = 9/22 (40.9%)	*p* Value
Diameter main MTC focus median (IQR, interval) (cm)	0.65 (0.25–1.05, 0.1–6.5)	0.40 (0.23–0.58, 0.10–0.60)	0.036
Multifocality	39 (65%)	6 (66%)	0.392
Bilaterality	17 (45%)	1 (15%)	0.676
T score more than 1	8 (14%)	0%	0.289
Lymph node metastasis at surgery	22 (38.6%)	0%	0.045
Distant metastasis at surgery	1 (1.85%)	0%	1.000

## Data Availability

The data presented in this study are available on request from the corresponding author. The data are not publicly available due to ethical issues.
